# Early career investigator biocommentary: Lauren Erdman

**DOI:** 10.1038/s41390-024-03216-1

**Published:** 2024-04-23

**Authors:** Lauren Erdman

**Affiliations:** Cincinnati Children’s Hospital Medical Center, 3333 Burnet Ave, Cincinnati, OH 45229 USA


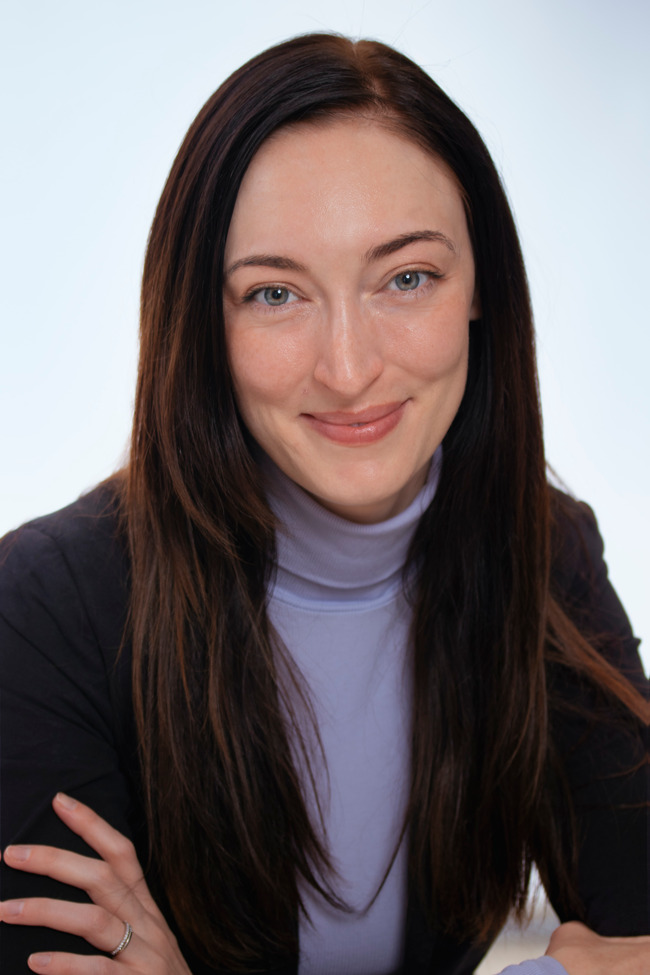


I am an assistant professor specializing in machine learning for biomedical applications at Cincinnati Children’s Hospital Medical Center and the University of Cincinnati College of Medicine in the James M. Anderson Center for Health Systems Excellence and the Division of Gastroenterology, Hepatology & Nutrition. I grew up in a small university town in Idaho state and moved to Vancouver, Canada to do my undergraduate training at Simon Fraser University (SFU). At SFU, I studied economic development and minored in statistics while working as a behavioral interventionist for children on the autistic spectrum. The combination of my studies and work taught me many things, including how pivotal early identification and intervention in pediatric conditions are on both the micro- and macro-scale, as well as how essential data is for making this possible. Therefore, when my undergraduate studies finished, I started my graduate studies in biostatistics at the University of Toronto (UofT).

In biostatistics, I focused on statistical genetics and had the opportunity to work in a pediatric psychiatric genetics lab at the Hospital for Sick Children in Toronto. Here I learned how evermore computational approaches to interrogating biomedical data enable our understanding and treatment of disease. My next step was honing my skills in computational modeling by pursuing a MSc and then a PhD in computer science at UofT, under the supervision of Dr. Anna Goldenberg. In my studies and through many collaborations, I was exposed to the wider world of computational biomedical analysis. I learned how essential team science is to impactful and novel discovery. Indeed, this lesson is reflected in all the studies coming from the Canadian Healthy Infant Longitudinal Disease study birth cohort, one of which I had the privilege to lead and is published in this issue of Pediatric Research, entitled *Early prediction of pediatric asthma in the Canadian Healthy Infant Longitudinal Development (CHILD) birth cohort using machine learning*.

My advice to early career researchers:

- Find good mentors, colleagues, and collaborators at every level who you can work alongside and learn from.
- Be opportunistic. Balance digging into established methods with exploring what’s on the horizon.
- Do work you enjoy. Research can be very difficult, demanding, discouraging, and slow at times. It’s much easier to persevere when you enjoy the topic, the process, and the people you work with.

